# Non‐canonical Raf‐1/p70S6K signalling in non–small‐cell lung cancer

**DOI:** 10.1111/jcmm.14636

**Published:** 2019-09-21

**Authors:** Zhixin Qiu, Bingwei Ye, Shuang Zhao, Xin Li, Lei Li, Ximing Mo, Weimin Li

**Affiliations:** ^1^ Department of Respiratory and Critical Care Medicine West China Hospital Sichuan University Chengdu China; ^2^ Georgia Cancer Center Augusta University Augusta GA USA; ^3^ Department of Biochemistry and Molecular Biology Medical College of Georgia Augusta University Augusta GA USA; ^4^ Laboratory of Stem Cell Biology State Key Laboratory of Biotherapy West China Hospital Sichuan University Chengdu China

**Keywords:** non‐canonical, non–small‐cell lung cancer, p70S6K, Raf‐1, signaling

## Abstract

Lung cancer is the leading cause of cancer‐related death globally, with non–small‐cell lung cancer (NSCLC) being the predominant subtype. Overall survival remains low for NSCLC patients, and novel targets are needed to improve outcome. Raf‐1 is a key component of the Ras/Raf/MEK signalling pathway, but its role and downstream targets in NSCLC are not completely understood. Our previous study indicated a possible correlation between Raf‐1 levels and ribosomal protein S6 kinase (p70S6K) function. In this study, we aimed to investigate whether p70S6K is a downstream target of Raf‐1 in NSCLC. Raf‐1 was silenced in NSCLC cell lines by using small hairpin RNA, and Raf‐1 and p70S6K protein levels were measured via Western blot. p70S6K was then overexpressed following Raf‐1 knock‐down; then, cell proliferation, apoptosis and the cell cycle in NSCLC cell lines were examined. Tumour xenografts with NSCLC cells were then transplanted for in vivo study. Tumours were measured and weighed, and Raf‐1 and p70S6K expression, cell proliferation and apoptosis were examined in tumour tissues by Western blot, Ki‐67 staining and TUNEL staining, respectively. When Raf‐1 was silenced, p70S6K protein levels were markedly decreased in the A549 and H1299 NSCLC cell lines. A significant decrease in NSCLC cell proliferation, a profound increase in apoptosis and cell cycle arrest were observed in vitro following Raf‐1 knock‐down. Overexpression of p70S6K after Raf‐1 depletion effectively reversed these effects. Xenograft studies confirmed these results in vivo. In conclusion, Raf‐1 targets p70S6K as its downstream effector to regulate NSCLC tumorigenicity, making Raf‐1/p70S6K signalling a promising target for NSCLC treatment.

## 1 INTRODUCTION

Lung cancer is the leading cause of cancer‐related death throughout the world.^1^ It is further classified into two subtypes: small‐cell lung cancer and non–small‐cell lung cancer (NSCLC), with NSCLC accounting for 85%‐90% of all lung cancer cases.^1^ Although NSCLC patients have benefited from advances in radiation therapy, targeted therapies and immunotherapies in recent years,^2^ only marginal improvement on the overall survival rate has been observed. The 5‐year survival rate for NSCLC patients remains as low as around 16%,^1^ and most patients die from metastasis and recurrence of the tumour. Therefore, novel targets and more efficient therapeutic approaches are urgently needed to improve the survival of NSCLC patients.

The Ras/Raf/MEK (mitogen‐activated protein kinase kinase) signalling pathway is a well‐known pathway involved in NSCLC. Its key component, Raf, a serine/threonine‐protein kinase, has three isoforms: A‐Raf, B‐Raf and Raf‐1 (C‐Raf). While B‐Raf is a well‐recognized oncogene whose mutation has been reported in NSCLC,^3^ the role of Raf‐1 in NSCLC development is considered essential as well,^4^ and elevated Raf‐1 expression in NSCLC has been previously reported.^5^ In addition, Raf‐1 has been shown to be an independent prognostic factor of NSCLC, and its high expression is associated with poor prognosis.^6^

Despite the intensive study focusing on Raf‐1 since its discovery, its complete role and substrates in NSCLC have not been fully elucidated. The classic Raf/MEK/ERK (extracellular signal‐regulated kinase) signalling is generally accepted as responsible for the function of Raf‐1 in cancer development, including in NSCLC. However, the fact that Raf‐1 is able to facilitate tumour development through mechanisms other than MEK/ERK signalling is evident by identification of other Raf‐1 targets.^7^ Ribosomal protein S6 kinase (p70S6K) is a well‐characterized downstream target of the mammalian target of rapamycin (mTOR) for its role in protein biosynthesis. Our previous observation suggested that the function of p70S6K might be correlated with Raf‐1 expression,^6^ which raises the likelihood of p70S6K being a downstream target of Raf‐1 in NSCLC. To this end, this study investigated the connection between Raf‐1 and p70S6K in NSCLC.

## 2 METHODS

### 2.1 Cell culture

Human NSCLC cell lines used in this study were obtained from the cell bank of the typical culture preservation committee of the Chinese Academy of Sciences. The cells were cultured in PRMIRPMI‐1640 (HyClone) or DMEM (HyClone) supplemented with 10% foetal bovine serum (HyClone) and 1% penicillin/streptomycin (HyClone), and were prior to being incubated at 37°C with 5% CO2 in a humidified incubator.

### 2.2 Overexpression and shRNA construct preparation

The p70S6K‐expressing construct GV287‐Ubi‐MCS‐3FLAG‐SV40‐EGFP‐p70S6K was generated by subcloning PCR‐amplified full‐length human p70S6K cDNA into the GV287‐Ubi‐MCS‐3FLAG‐SV40‐EGFP vector (GeneChem) following standard protocol. Raf‐1–specific shRNA and negative control were designed using BLOCK‐iT (Invitrogen), and DNA oligonucleotides were synthesized by GeneChem. Complementary oligonucleotides were suspended in annealing buffer. The oligonucleotide mixture was then heated to 90°C for 15 minutes and gradually cooled down to room temperature. The Raf‐1–specific shRNA construct GV197‐shRaf1‐hU6‐MCS‐CMV‐cherry and the negative control construct GV197‐NC‐hU6‐MCS‐CMV‐cherry were generated by introducing annealed shRNA oligonucleotides into GV197‐hU6‐MCS‐CMV‐cherry vector (GeneChem) according to standard subcloning protocols. Correct constructs were verified by sequencing.

Designed shRNA oligonucleotides:

**Table:** 

shRaf‐1	5′	STEM	Loop	STEM	3′
Sense	Ccgg	gaGACATGAAATCCAACAATA	CTCGAG	TATTGTTGGATTTCATGTCTC	TTTTTg
Antisense	aattcaaaaa	gaGACATGAAATCCAACAATA	CTCGAG	TATTGTTGGATTTCATGTCTC	

**Table:** 

NC	5′	STEM	Loop	STEM	3′
Sense	T	TTCTCCGAA CGTGTCACGT	TTCAAGA GA	ACGTGACACGTT CGGAGAA	TTTT TTC
Antisense	TCGAGA AAAAA	TTCTCCGAAC GTGTCACGT	TCTCTT GAA	ACGTGACACG TTCGGAGAA	A

### 2.3 Overexpression and RNA interference

Lentiviral constructs expressing p70S6K and shRNA targeting Raf‐1 were transfected into 293T cells along with packaging plasmids pGC‐LV, pHelper 1.0 and pHelper 2.0 (GeneChem) using Lipofectamine 2000 reagent (Invitrogen Life Technologies) for lentivirus production. Lentivirus‐containing supernatants were harvested to infect target cells with 5 μg/mL polybrene. Lentivirus‐infected NSCLC cells were sorted using a BD FACSCalibur flow cytometer (BD Biosciences).

### 2.4 Western blot analysis

Proteins were extracted from cells using a protein extraction kit (KeyGEN), and protein concentrations were measured using a BCA protein assay kit (Pierce). The proteins were then separated on sodium dodecyl sulphate‐polyacrylamide (SDS‐PAGE) gels, transferred to a polyvinylidene fluoride (PVDF) membrane (Millipore) and probed with specific primary antibodies overnight, including anti–Raf‐1 (Cell Signalling Technology), anti‐p70S6K (Cell Signalling Technology) and the internal control anti–β‐actin (Cell Signalling Technology) or anti‐GAPDH (Santa Cruz Biotechnology). Horseradish peroxidase‐conjugated goat anti‐rabbit IgG (Cell Signalling Technology) was used as the secondary antibody. The target proteins were visualized using the Luminata Crescendo Western HRP substrate Kit (Millipore), and the integrated optical density (IOD) of target bands was quantified using Image‐Pro Plus software (Media Cybernetics).

### 2.5 Cell proliferation assay

Cell proliferation was assessed by using the Cell Counting Kit‐8 assay (CCK‐8; Dojindo). Briefly, cells were seeded into 96‐well culture plates at a density of 2 × 10^3^ cells/well. Culture media was replaced with 10% CCK‐8 at 24, 72 and 120 hours after initial seeding. The cells were then incubated at 37°C for 1 hour, and absorbance was subsequently measured at a wavelength of 450 nm.

### 2.6 Cell apoptosis assay

Harvested cells were washed with PBS and resuspended in 1 × binding buffer. The cells were labelled with Annexin‐V‐FITC or Annexin‐V‐APC and then stained with DAPI. Annexin‐V‐positive and DAPI‐negative cells were counted as apoptotic cells. A FACSCalibur flow cytometer (Becton Dickinson) was used to acquire data, which were analysed by using Navios platform system software (Beckman Coulter).

### 2.7 Cell cycle assay

The cells were harvested and fixed in 70% cold ethanol at 4°C overnight. A mixture of 50 μg/mL propidium iodide (PI; JingBo), 100 μg/mL RNAase and 0.2% Triton X‐100 was applied for staining. DNA content data were acquired by flow cytometry, and cell cycle distributions were analysed using ModFit software (Verity Software House).

### 2.8 Tumour xenograft

Female nude mice (4‐6 weeks old) were used to host xenograft tumours. 1 × 10^6^ NSCLC cells in 100 μL PBS were injected subcutaneously into the right flanks of recipient mice. Tumour sizes were measured, and the experiment was terminated 3‐4 weeks after injection. Tumours were then harvested, weighed and saved for Western blot analysis, TUNEL assay and immunohistochemical staining with Ki‐67.

### 2.9 TUNEL assay

Paraffin‐embedded tumour tissues were sectioned into 4 μm thickness for staining. The In Situ Cell Death Detection Kit, Fluorescein (Roche Diagnostics GmbH), was used to apply TUNEL staining to tumour tissues in the shRaf‐1 group, and the nuclei of apoptotic tumour cells showed green fluorescence. FragEL^™^ DNA Fragmentation Detection Kit, Colorimetric‐TdT Enzyme (MERCK), was used for TUNEL staining on tumour tissues in the negative control and shRaf‐1 plus p70S6K overexpression groups. Apoptotic cells in these groups showed brown nuclei.

### 2.10 Immunohistochemical staining

Ki‐67 staining was performed on 4‐μm‐thick paraffin‐embedded tissue sections. Tissue sections were incubated with anti‐Ki‐67 primary antibody (1:100 dilution; Dako) overnight at 4°C, and then, secondary antibody was applied. 3′‐Diaminobenzidine (DAB) was used as a chromogen substrate.

### 2.11 Statistical analysis

Data are presented as the mean ± standard deviation and were analysed by unpaired, two‐tailed Student's *t* test or two‐way ANOVA. Statistical analysis was performed using SPSS 17.0 software or GraphPad Prism 7.0a, and *P* < .05 was considered statistically significant.

## 3 RESULTS

### 3.1 Raf‐1 regulates p70S6K protein expression

**Figure 1:**
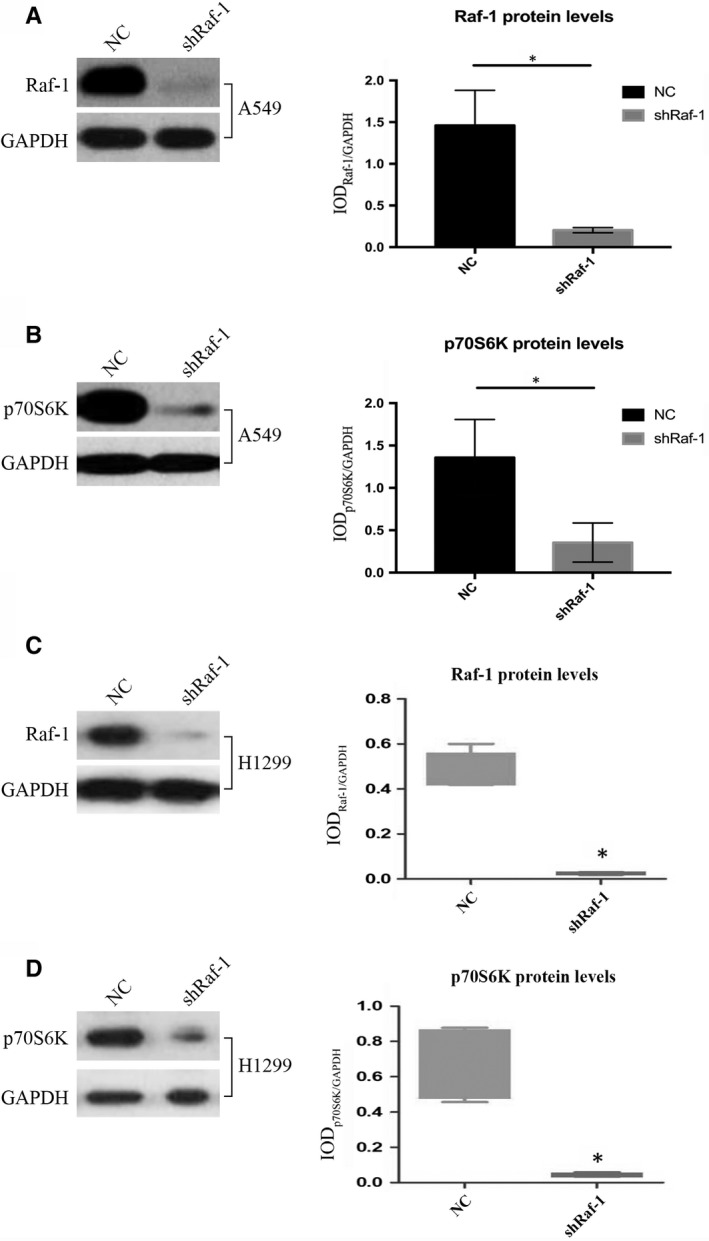
Raf‐1 regulates p70S6K protein expression. A, Western blot analysis examining Raf‐1 protein expression in response to Raf‐1 knock‐down in the A549 NSCLC cell line. GAPDH was used as a loading control. Raf‐1 protein levels were quantified and are expressed as IOD of Raf‐1/GAPDH (6 biological replicates). B, Western blot analysis examining p70S6K protein levels following Raf‐1 knock‐down in the A549 NSCLC cell line. GAPDH was used as a loading control. The p70S6K protein level was quantified and expressed as the IOD of p70S6K/GAPDH (6 biological replicates). C. Western blot analysis examining Raf‐1 protein expression in response to Raf‐1 knock‐down in the H1299 NSCLC cell line. GAPDH was used as a loading control. Raf‐1 protein levels were quantified and are expressed as IOD of Raf‐1/GAPDH. D. Western blot analysis examining p70S6K protein levels following Raf‐1 knock‐down in the H1299 NSCLC cell line. GAPDH was used as a loading control. The p70S6K protein level was quantified and expressed as the IOD of p70S6K/GAPDH. *P <* *.05

We initially examined the correlation between Raf‐1 and p70S6K protein levels. Raf‐1 was silenced with small hairpin RNA (shRNA) in the A549 and H1299 NSCLC cell lines, and its protein expression levels were examined via Western blot analysis. A significant decrease in Raf‐1 protein levels was observed in both cell lines compared to the negative controls (Figure 1A and 1C). Furthermore, in both cell lines, with the depletion of Raf‐1, protein levels of p70S6K were correspondingly reduced (Figure 1B and 1D), indicating that Raf‐1 regulates the expression of p70S6K in NSCLC.

### 3.2 Raf‐1 functions through p70S6K to sustain NSCLC cell proliferation

**Figure 2:**
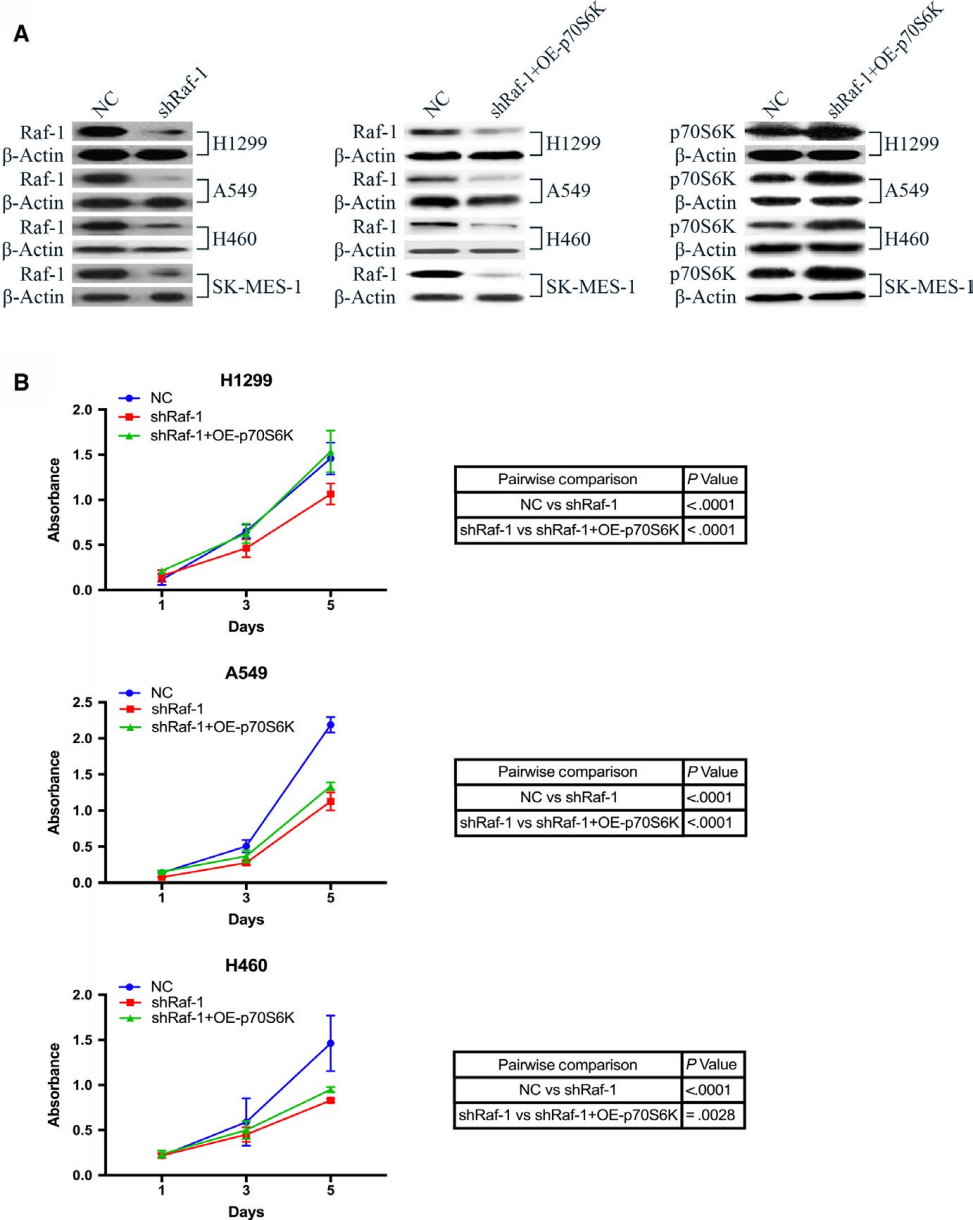
Raf‐1 functions through p70S6K to sustain NSCLC cell proliferation. A, Western blot analysis confirming Raf‐1 knock‐down and p70S6K overexpression in all NSCLC cell lines (H1299, A549, H460 and SK‐MES‐1) used in this study. β‐Actin levels were used as a loading control. B, Growth curve of NSCLC cell lines H1299 (top), A459 (middle) and H460 (bottom) with negative control (NC), Raf‐1 knock‐down (shRaf‐1), and p70S6K overexpression following Raf‐1 knock‐down (shRaf‐1 + OE‐p70S6K)

We next examined the effect of Raf‐1 knock‐down on NSCLC cell proliferation. A profound decrease in cell proliferation of H1299, A549 and H460 NSCLC cells was observed (Figure 2B, shRaf‐1) in response to silencing Raf‐1 with shRNA, demonstrating the essential role of Raf‐1 in NSCLC cell proliferation. Given that p70S6K protein expression was repressed following Raf‐1 ablation, we further tested whether introducing exogenous p70S6K after Raf‐1 depletion rescues NSCLC cell growth. Overexpression of p70S6K following Raf‐1 knock‐down significantly reversed the proliferation‐suppressing effects of Raf‐1 depletion (Figure 2B, shRaf‐1 + OE‐p70S6K), indicating that p70S6K mediates the critical effect of Raf‐1 in sustaining NSCLC cell proliferation.

### 3.3 Raf‐1 signals through p70S6K to prevent NSCLC cell from undergoing apoptosis

**Figure 3:**
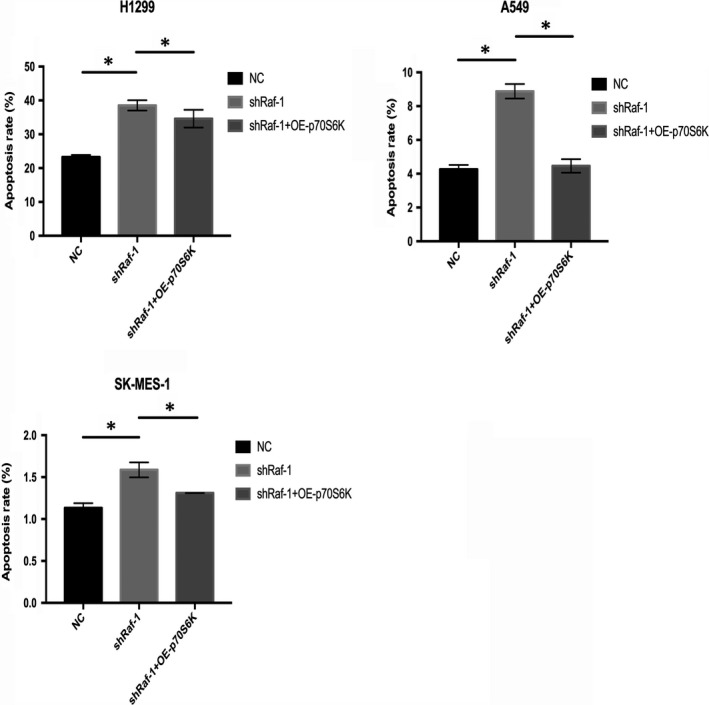
Raf‐1 signals through p70S6K to prevent apoptosis in NSCLC cells. Apoptosis rates of H1299, A549 and SK‐MES‐1 NSCLC cell lines with negative control (NC), Raf‐1 knock‐down (shRaf‐1), and p70S6K overexpression following Raf‐1 knock‐down (shRaf‐1 + OE‐p70S6K). *P <* *.05

NSCLC cell apoptosis was assessed next. We found that the apoptosis rate of NSCLC cells was markedly increased in response to Raf‐1 silencing (Figure S1). The apoptosis rates of H1299, A549 and SK‐MES‐1 NSCLC cell lines were significantly elevated in response to Raf‐1 knock‐down compared to negative controls (Figure 3, shRaf‐1 vs NC). Then, we overexpressed p70S6K following Raf‐1 depletion. The apoptosis‐inducing effect caused by Raf‐1 silencing was largely reversed by p70S6K overexpression (Figure 3, shRaf‐1 + OE‐p70S6K vs shRaf‐1). These results suggest that Raf‐1 exerts its function through p70S6K to inhibit NSCLC cell apoptosis.

### 3.4 Raf‐1 promotes cell cycle progression of NSCLC via p70S6K

**Figure 4:**
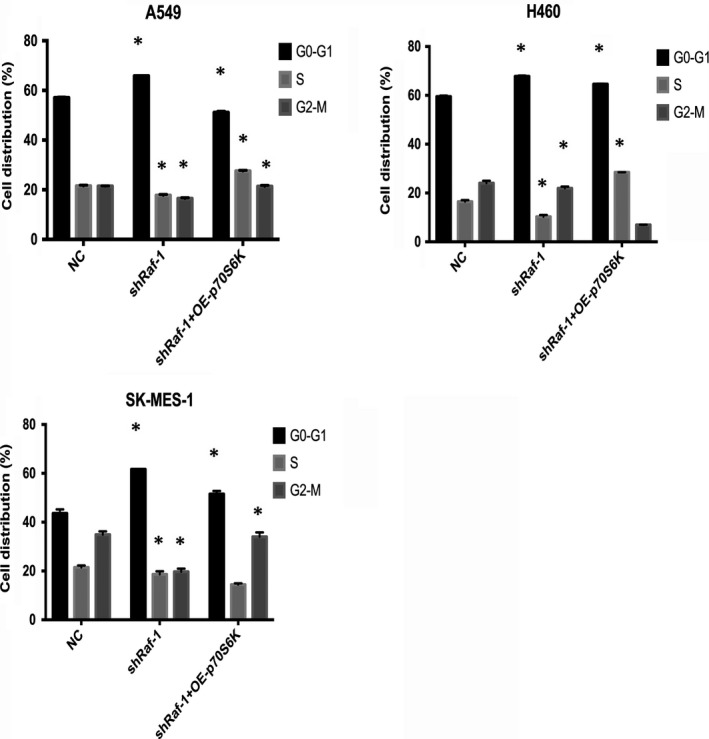
Raf‐1 promotes cell cycle progression of NSCLC via p70S6K. Cell cycle distributions of A549, H460 and SK‐MES‐1 NSCLC cell lines with negative control (NC), Raf‐1 knock‐down (shRaf‐1) and p70S6K overexpression following Raf‐1 knock‐down (shRaf‐1 + OE‐p70S6K). *P <* *.05

Similar results were found for NSCLC cell cycle progression (Figure S2). When Raf‐1 was silenced, we observed cell cycle arrest in NSCLC cells with a profound increase in G0‐G1 phase and a marked decrease in S and G2‐M phases (Figure 4, shRaf‐1 vs NC). Overexpression of p70S6K following Raf‐1 knock‐down resulted in significantly more NSCLC cells entering the cell cycle and progressing into S/G2‐M phases than that observed with Raf‐1 ablation (Figure 4, shRaf‐1 + OE‐p70S6K vs shRaf‐1). Taken together, these results demonstrate that p70S6K mediates Raf‐1 signalling to facilitate the G1 phase transition in NSCLC cells.

### 3.5 Raf‐1/p70S6K signalling maintains NSCLC tumorigenicity in vivo

**Figure 5:**
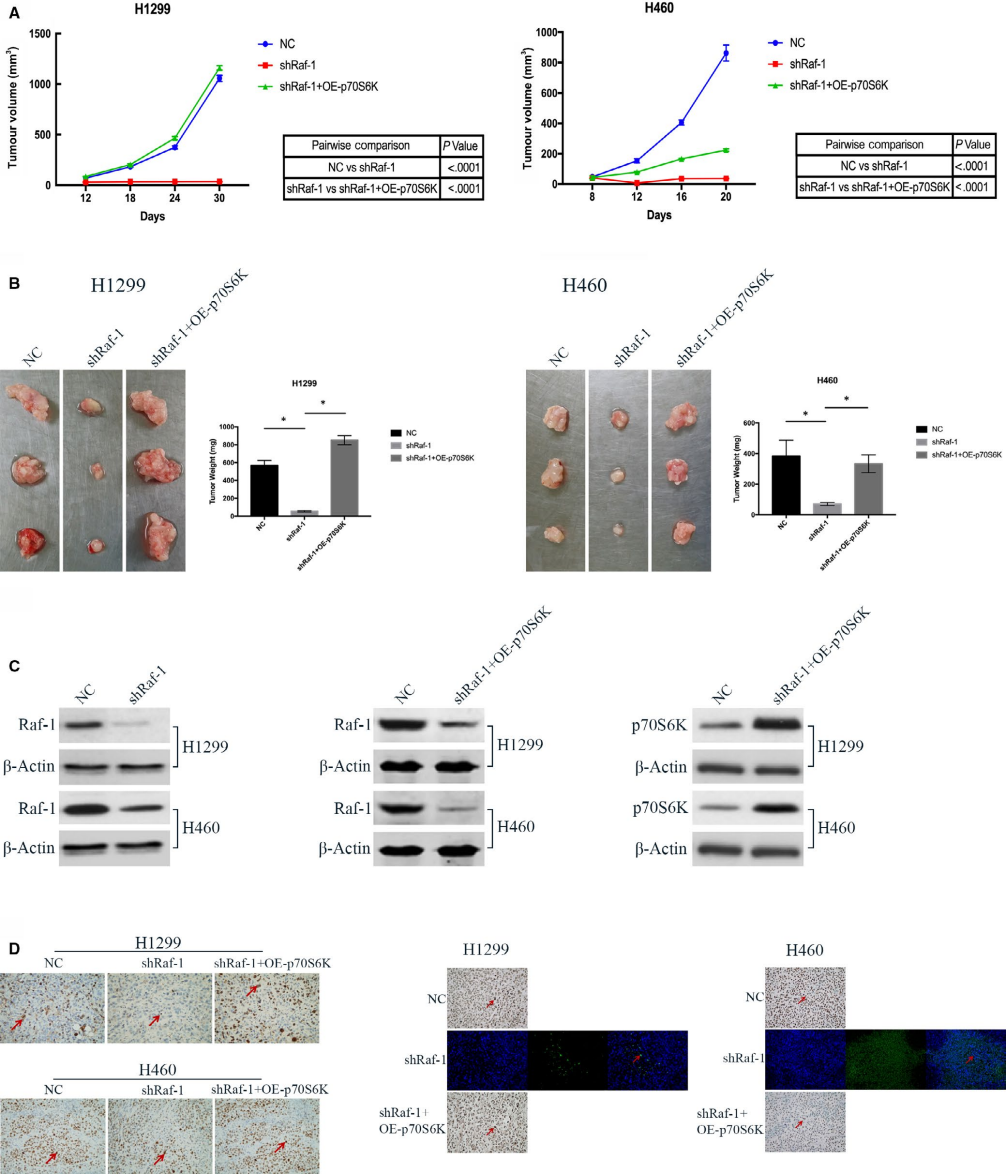
Raf‐1/p70S6K signalling maintains NSCLC tumorigenicity in vivo. A, Tumour growth curve of tumours generated from H1299 (left) and H460 (right) cells with negative control (NC), Raf‐1 knock‐down (shRaf‐1) and p70S6K overexpression following Raf‐1 knock‐down (shRaf‐1 + OE‐p70S6K). B, Comparison of tumour weights between negative control (NC) and Raf‐1 knock‐down (shRaf‐1) and between Raf‐1 knock‐down (shRaf‐1) and p70S6K overexpression following Raf‐1 knock‐down (shRaf‐1 + OE‐p70S6K) groups; *P < .05. C, Western blot analysis confirming the knock‐down status of Raf‐1 and the overexpression status of p70S6K in tumour tissues. D, Immunohistochemical staining with Ki‐67 (left) showing NSCLC tumour cell proliferation in each group (×400). Large brown Ki‐67 staining (arrow) indicates proliferating cells. TUNEL staining (middle and right) demonstrating apoptotic cells in each group (×400). Positive cells are indicated by arrows

We further conducted in vivo experiments using xenograft mouse models established with the H1299 and H460 NSCLC cell lines. Similar to our in vitro observations, depletion of Raf‐1 led to profound tumour growth arrest (Figure 5A, shRaf‐1), whereas overexpression of p70S6K abolished this growth arrest (Figure 5A, shRaf‐1 + OE‐p70S6K), which was further confirmed by comparing tumour weights between these two groups (Figure 5B). Analysis of tumour tissues by Western blot confirmed reduced Raf‐1 expression and elevated p70S6K protein levels within the tumours (Figure 5C). Moreover, tumour tissues in Raf‐1 knock‐down groups showed suppressed proliferation and increased apoptosis, as demonstrated by Ki‐67 and TUNEL staining, respectively. On the other hand, overexpression of p70S6K resulted in an obvious reverse of both the proliferation‐inhibiting and apoptosis‐inducing effects (Figure 5D). Together, these results suggest that Raf‐1 signals through p70S6K to exert its pivotal role in NSCLC tumorigenicity, further confirming that p70S6K is a *bona fide* downstream target of Raf‐1.

## 4 DISCUSSION

**Figure 6:**
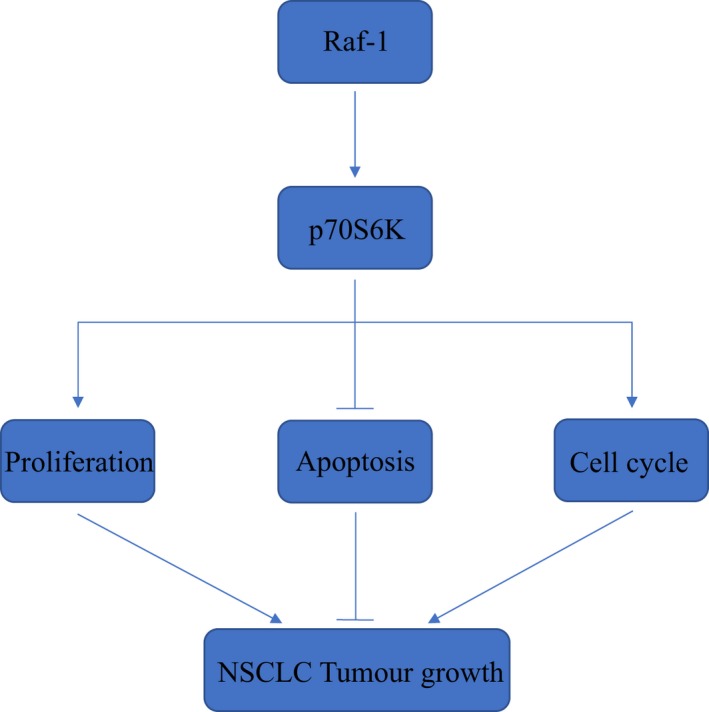
Schematic diagram illustrating the Raf‐1/p70S6K signalling pathway in NSCLC. Raf‐1 signals through p70S6K to sustain proliferation, inhibit apoptosis and promote cell cycle progression of NSCLC cells, leading to NSCLC tumour growth

In this study, we identified a non‐canonical Raf‐1/p70S6K signalling pathway in NSCLC where Raf‐1 targets p70S6K as its downstream effector to regulate NSCLC tumour growth via sustaining proliferation, inhibiting apoptosis and promoting cell cycle progression (Figure 6).

The Ras/Raf/MEK signalling pathway is pivotal in NSCLC. Aberrant activation of this pathway and mutations of its key components, especially K‐Ras and B‐Raf, frequently occur in NSCLC.^1^, ^3^, ^8^ While Raf‐1 mutations are rare in this devastating disease, Raf‐1 expression is often abnormally elevated in NSCLC.^5^ Notably, Raf‐1 is a key mediator of NSCLC; Raf‐1 is so far the only known mediator in the Raf family and is responsible for initiation and development of K‐Ras mutation‐driven NSCLC.^4^, ^9^ In addition, our previous study revealed that Raf‐1 is an independent prognostic factor for NSCLC, and a positive correlation exists between elevated Raf‐1 expression levels and poor prognosis of NSCLC patients,^6^ also implying the essential role of Raf‐1 in NSCLC. Consistent with previous studies demonstrating the important role of Raf‐1 in NSCLC, our current findings confirmed that Raf‐1 is essential in regulating NSCLC tumorigenicity both in vitro and in vivo. The resultant suppression of proliferation, induction of apoptosis and cell cycle arrest due to Raf‐1 depletion adds further evidence in support of the critical role for Raf‐1 in NSCLC.

We also provide evidence of a connection between Raf‐1 and p70S6K by identifying the Raf‐1/p70S6K signalling pathway in NSCLC. This finding is in accordance with a previous study that linked Raf‐1 and p70S6K together in liver cancer.^10^ The mechanism of how Raf‐1 and p70S6K interact with each other in NSCLC, however, remains obscure. MEK is a classic downstream effector of Raf‐1 and is generally involved in the majority of Raf‐1 functions. According to Leung et al,^10^ MEK also mediates signalling from Raf‐1 to p70S6K in human liver cancer cells to form a Raf‐1/MEK/p70S6K signalling pathway. Hence, it is possible that MEK plays the same role in connecting Raf‐1 and p70S6K in NSCLC as that in liver cancer. Nevertheless, MEK is not required for all Raf‐1 functions. For example, MEK kinase activity is not necessary for Raf‐1–mediated mouse development.^11^ In addition, Raf‐1 directly interacts with Bcl‐2 (B‐cell lymphoma 2),^7^ ASK1 (apoptosis signal‐regulating kinase 1)^12^ and MST2 (serine/threonine‐protein kinase 3)^13^ proteins to inhibit apoptosis in a MEK‐independent manner. The MEK‐independent function of Raf‐1 is also evident by the discovery that Raf‐1 ablation induces NSCLC regression via a mitogen‐activated protein kinase (MAPK) signalling‐independent mechanism.^14^ Moreover, it has been demonstrated that Raf‐1 activates p70S6K independent of MAPK signalling based on observations that not all MAPK agonists were able to activate p70S6K, that blockage of MAPK activation would not affect p70S6K stimulation and that an oestradiol‐regulated form of Raf‐1 still activates p70S6K in CCL39 lung fibroblasts under conditions of blocked MAPK activation.^15^ Therefore, the possibility of Raf‐1 directly interacting with p70S6K independent of MEK in NSCLC cannot be ruled out, and further investigation is needed to elucidate the underlying mechanism of the interaction between Raf‐1 and p70S6K in NSCLC.

Another important signalling pathway in NSCLC is the PI3K (phosphatidylinositol 3‐kinase)/Akt (protein kinase B)/mTOR signalling pathway. Both Ras/Raf/MEK and PI3K/Akt/mTOR pathways are involved in numerous physiological and pathological processes. In general, these two classic pathways are involved in regulating cell growth, proliferation, differentiation, apoptosis, metabolism and motility to maintain homoeostasis under physiological conditions.^16^ Further, the aberrant activation of at least one of these two pathways has been observed in various diseases, particularly in cancers, such as breast cancer,^17^ prostate cancer^18^ and NSCLC.^19^ Crosstalk between these two pathways adds another layer of complexity to this network. For instance, the mTOR upstream activator Rheb (RAS homologue enriched in brain) inhibits both Raf‐1^20^ and B‐Raf.^21^ Negative regulation also includes phosphorylation of GAB (GRB2‐associated binder) by ERK, which inhibits GAB‐mediated PI3K recruitment.^16^ Positive regulations exist in this network as well. By regulating PI3K, TSC2 (tuberous sclerosis complex 2) and mTOR, Ras, Raf and ERK can cross‐activate the PI3K/Akt/mTOR pathway.^16^ Another form of crosstalk between these two pathways involves pathway convergence. ERK and Akt, for example, both target the same substrate, FOXO3A (forkhead box O3A).^16^ Herein, by identifying Raf‐1/p70S6K signalling, we have identified another pathway convergence in this network as the mutual effector p70S6K of both mTOR and Raf‐1.

Lung cancer is the leading cause of cancer‐related death globally, and the overall survival of NSCLC remains dismal. Current treatments include surgery, chemotherapy, radiotherapy, radiofrequency ablation, immunotherapy and molecular targeted therapy.^22^, ^23^ Patients in advanced stages with driving mutations benefit most from targeted therapy. Majority of US Food and Drug Administration (FDA)‐approved medications in clinical use for NSCLC treatment are those targeting epidermal growth factor receptor (EGFR) mutations (EGFR tyrosine kinase inhibitors), anaplastic lymphoma kinase (ALK) and ROS1 proto‐oncogene receptor tyrosine kinase (ROS1) fusions (ALK inhibitors), and B‐Raf mutations.^24^ Nevertheless, incomplete and temporary responses to these medications are generally observed, and resistances frequently occur due to intrinsic, adaptive or acquired mechanisms, which either alter the primary target of a certain agent or use collateral signalling pathways instead to bypass the requirement of the driver oncoproteins, hence leading to insensitivity to the medications.^24^ Novel targets are needed for NSCLC treatment; therefore, identification of this non‐canonical Raf‐1/p70S6K pathway may shed light on therapeutic strategies for NSCLC. K‐Ras mutation accounts for roughly 25% of NSCLC;^25^ however, K‐Ras cannot be directly targeted for treatment.^9^ Hence, targeting downstream K‐Ras substrates may represent to be a more feasible approach. Raf‐1 is the only mediator in the Raf family to transduce K‐Ras signals in NSCLC for tumorigenicity,^4^, ^9^ and Raf‐1 ablation has limited toxicity,^14^ making Raf‐1 an attractive therapeutic target for K‐Ras–driven NSCLC. However, this approach might still be challenging given the allosteric function of Raf‐1, through which drug‐bound inactivated Raf‐1 can bind to and activate drug‐free Raf‐1.^7^ Therefore, targeting downstream effectors of Raf‐1 appears to be a wise option. Unfortunately, systemic MEK and ERK ablation is correlated with intolerant toxicity.^14^ Hence, other Raf‐1 downstream targets might be more suitable for targeted therapy than MEK or ERK. Additionally, the PI3K/Akt/mTOR pathway is also altered in NSCLC, especially in squamous cell lung cancer;^26^ the crosstalk between Ras/Raf/MEK and PI3K/Akt/mTOR signalling pathways makes NSCLC treatment even more challenging, since inhibiting one pathway may lead to corresponding activation of the other due to blocking negative regulation. In this case, p70S6K may represent an excellent target because it serves as a mutual downstream effector of Raf‐1 and mTOR. Furthermore, our previous study showed effective inhibition on NSCLC tumorigenicity with a p70S6K‐specific inhibitor, PF‐4708671,^27^ indicating that p70S6K is a promising target for therapeutic intervention in NSCLC. Therefore, our current identification of the Raf‐1/p70S6K signalling pathway may lead to a new avenue in NSCLC therapy.

In conclusion, we identified a Raf‐1/p70S6K signalling pathway in NSCLC. This pathway is responsible for NSCLC tumorigenicity by regulating tumour cell proliferation, apoptosis and cell cycle progression. This finding reveals further crosstalk between Ras/Raf/MEK and the PI3K/Akt/mTOR signalling pathways and suggests that the Raf‐1/p70S6K pathway, especially p70S6K, could be a promising target for NSCLC treatment.

## CONFLICT OF INTEREST

The authors declare no potential conflicts of interest.

## AUTHOR CONTRIBUTIONS

Z.Q., B.Y. and W.L. designed the study. Z.Q., S.Z. X.M. and L.L. performed the experiments and analysed the data. Z.Q. and B.Y. wrote the manuscript with contributions from X.L.
