# Corrigendum

**DOI:** 10.1111/jcmm.17587

**Published:** 2022-12-15

There are several errors in the Figure 2A and Figure 5D. In Figure 2A, the three WB strips framed in red need to be replaced. In Figure 5D, the NC group immunohistochemistry of H460 cell line which framed in red needs to be replaced. The authors confirm that all other results and conclusions of this article remain unchanged.

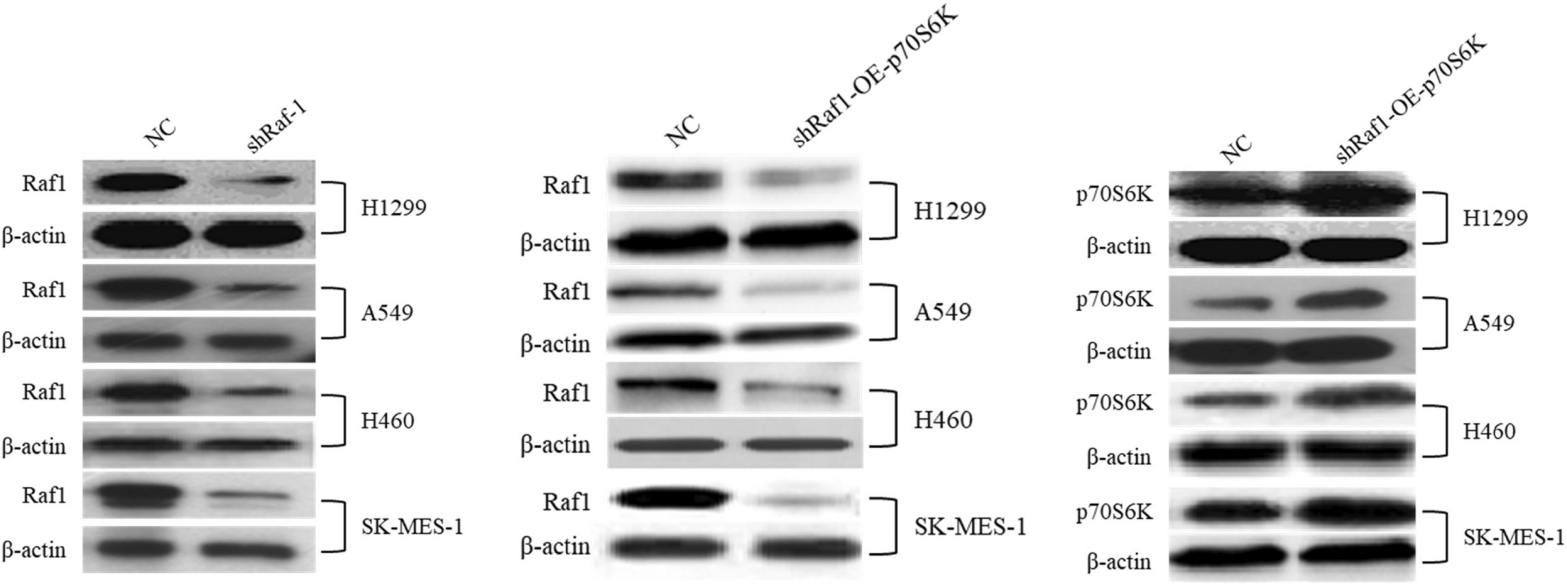

**FIGURE 2A**. Raf‐1 Functions through p70S6K to Sustain NSCLC Cell Proliferation.

A. Western blotting analysis confirming Raf‐1 knockdown and p70S6K overexpression in all NSCLC cell lines (H1299, A549, H460, and SK‐MES‐1) used in this study. β‐actin levels were used as loading control.

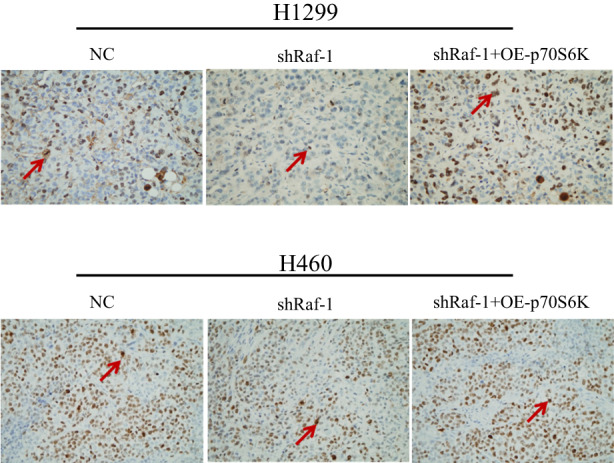

**FIGURE 5D**. Raf‐1/p70S6K Signalling Maintains NSCLC Tumorigenicity in vivo.

D. Immunohistochemical staining with Ki‐67 (left) showing NSCLC tumour cell proliferation in each group (×400). Large brown Ki‐67 staining (arrow) indicates proliferating cells. TUNEL staining (middle and right) demonstrating apoptotic cells in each group (×400); positive cells are indicated by arrows.

