# Supplementary figures and images for: Non‐canonical Raf‐1/p70S6K signalling in non–small‐cell lung cancer
Source: J Cell Mol Med. 2019 Sep 21;23(11):7632–40. doi: 10.1111/jcmm.14636 (PMC6815804; doi:10.1111/jcmm.14636)

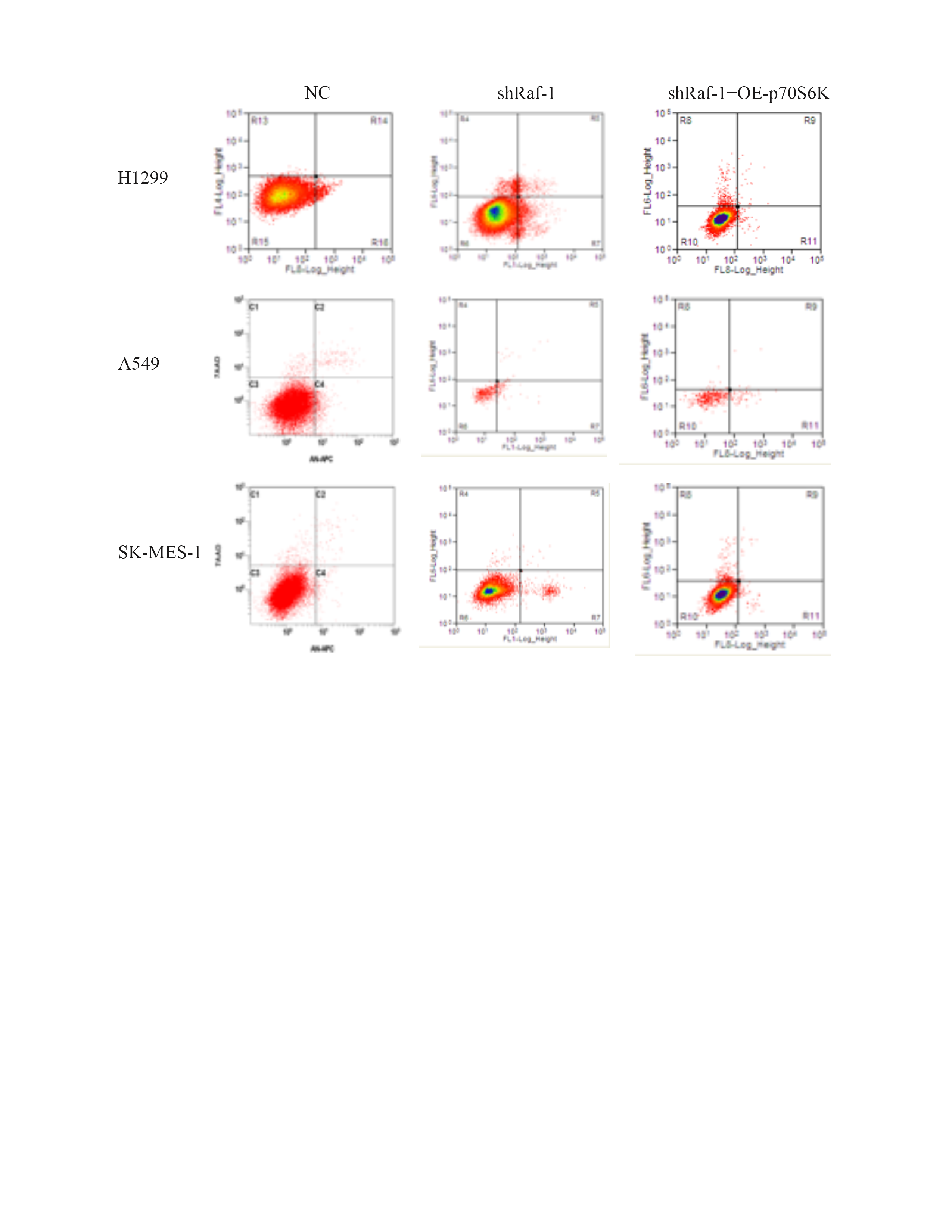

Supplement: Supplementary file 1 [file JCMM-23-7632-s001.tif]

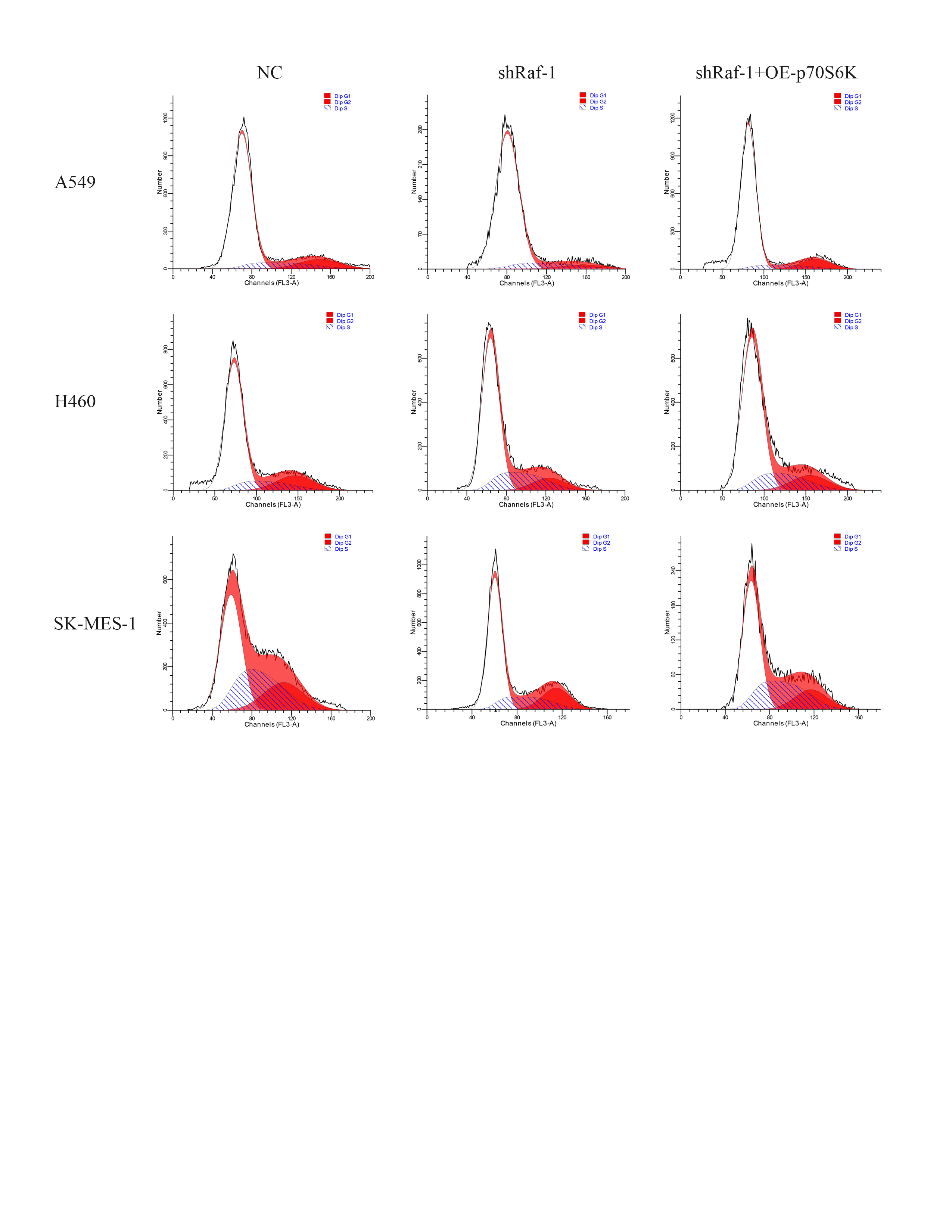

Supplement: Supplementary file 2 [file JCMM-23-7632-s002.tif]
